# Vandetanib in locally advanced or metastatic differentiated thyroid cancer refractory to radioiodine therapy

**DOI:** 10.1530/ERC-23-0354

**Published:** 2024-07-02

**Authors:** Marcia S Brose, Jaume Capdevila, Rossella Elisei, Lars Bastholt, Dagmar Führer-Sakel, Sophie Leboulleux, Iwao Sugitani, Matthew H Taylor, Zhuoying Wang, Lori J Wirth, Francis P Worden, John Bernard, Paolo Caferra, Raffaella M Colzani, Shiguang Liu, Martin Schlumberger

**Affiliations:** 1 Department of Medical Oncology, Sidney Kimmel Cancer Center at Thomas Jefferson University, Philadelphia, Pennsylvania, USA; 2 Gastrointestinal and Endocrine Tumor Unit, Medical Oncology Department, Vall d’Hebron University Hospital, Vall d’Hebron Institute of Oncology (VHIO), Barcelona, Spain; 3 Unit of Endocrinology, Department of Clinical and Experimental Medicine, University of Pisa, Pisa, Italy; 4 Department of Clinical Oncology, Odense University Hospital, Odense, Denmark; 5 Department of Endocrinology, Diabetes and Metabolism and Clinical Chemistry, University Hospital Essen, Essen, Germany; 6 Department of Nuclear Medicine and Endocrine Oncology, Gustave Roussy and Université Paris Saclay, Villejuif, France; 7 Department of Endocrinology, Diabetology, Nutrition and Therapeutic Education, Hôpitaux Universitaires de Genève, Geneve, Switzerland; 8 Department of Endocrine Surgery, Nippon Medical School Graduate School of Medicine, Tokyo, Japan; 9 Earle A. Chiles Research Institute, Providence Cancer Institute, Portland, Oregon, USA; 10 Department of Head Neck Surgery, Fudan University Shanghai Cancer Center, Shanghai, China; 11 Department of Head Neck Surgery, Renji Hospital Affiliated to Jiaotong University School of Medicine, Shanghai, China; 12 Department of Medicine, Massachusetts General Hospital and Harvard Medical School, Boston, Massachusetts, USA; 13 Rogel Cancer Center, University of Michigan, Ann Arbor, Michigan, USA; 14 Sanofi, Cambridge, Massachusetts, USA; 15 Sanofi, Amsterdam, The Netherlands; 16 Department of Pharmacy, University of Pisa, Pisa, Italy

**Keywords:** vandetanib, multikinase inhibitor, differentiated thyroid cancer, progression-free survival, radioiodine refractory

## Abstract

The VERIFY study aimed to determine the efficacy of vandetanib in patients with differentiated thyroid cancer (DTC) that is either locally advanced or metastatic and refractory to radioiodine (RAI) therapy. Specifically, VERIFY is a randomized, double-blind, multicenter phase III trial aimed to determine the efficacy and safety of vandetanib in tyrosine kinase inhibitor-naive patients with locally advanced or metastatic RAI-refractory DTC with documented progression (NCT01876784). Patients were randomized 1:1 to vandetanib or placebo. The primary endpoint was progression-free survival (PFS). Secondary endpoints included best objective response rate, overall survival (OS), safety, and tolerability. Patients continued to receive randomized treatment until disease progression or for as long as they were receiving clinical benefit unless criteria for treatment discontinuation were met. Following randomization, 117 patients received vandetanib, and 118 patients received a placebo. Median PFS was 10.0 months in the vandetanib group and 5.7 months in the placebo group (hazard ratio: 0.75; 95% CI: 0.55–1.03; *P* = 0.080). OS was not significantly different between treatment arms. Common Terminology Criteria for Adverse Events (CTCAE) of grade ≥3 were reported in 55.6% of patients in the vandetanib arm and 25.4% in the placebo arm. Thirty-three deaths (28.2%; one related to study treatment) occurred in the vandetanib arm compared with 16 deaths (13.6%; two related to treatment) in the placebo arm. No statistically significant improvement was observed in PFS in treatment versus placebo in patients with locally advanced or metastatic, RAI-refractory DTC. Moreover, active treatment was associated with more adverse events and more deaths than placebo, though the difference in OS was not statistically significant.

## Introduction

Differentiated thyroid cancer (DTC), including papillary, follicular, oncocytic carcinoma, and poorly differentiated cancers, arises from aberrant follicular cells of the thyroid and accounts for >90% of all thyroid malignancies (Sherman 2003, Haugen *et al.* 2016). Although most patients do well and are cured with surgery, with or without subsequent radioiodine (RAI) and – in high-risk patients – thyroid-stimulating hormone (TSH) suppression, some patients have a persistent disease that does not respond to treatment (Durante *et al.* 2006, Haugen *et al.* 2016, Naoum *et al.* 2018). About 10% of patients with DTC will develop distant metastases (Naoum *et al.* 2018), and two-thirds of these patients will be unresponsive to RAI (RAI refractory) (Durante *et al.* 2006). The global incidence of RAI-refractory DTC is approximately four to five new cases per year per million, and without additional treatment, the 10-year survival is less than 10%, with a median life expectancy of 3.5–5.5 years (Durante *et al.* 2006, Robbins *et al.* 2006, Schmidt *et al.* 2017).

Cytotoxic chemotherapy (e.g. doxorubicin) has limited efficacy and considerable toxicity in RAI-refractory DTC (Schmidt *et al.* 2017, Tumino *et al.* 2017). Therefore, multikinase inhibitors (MKIs) have been investigated as new treatments for patients with this rare disease. Currently, sorafenib and lenvatinib are approved in the USA, Europe, and Japan for the treatment of locally recurrent or metastatic, progressive RAI-refractory DTC (Brose *et al.* 2014, Schlumberger *et al.* 2015). Sorafenib targets rearranged during transfection (RET) receptor, vascular endothelial growth factor receptor (VEGFR) 1─3, proto-oncogene c-KIT, and proto-oncogene B-Raf (BRAF) (Schmidt *et al.* 2017). Lenvatinib targets VEGFR 1─3, fibroblast growth factor receptors (FGFR) 1─4, platelet-derived growth factor receptor-b (PDGFR-b), RET, and c-KIT (Schmidt *et al.* 2017). Of note, cabozantinib in second line (Brose *et al.* 2021), selpercatinib in case of RET fusion (Wirth *et al.* 2020), and larotrectinib (Waguespack *et al.* 2022) and entrectinib (Doebele *et al.* 2020) in case of a neurotrophic tyrosine receptor kinase (NTRK) fusion are also approved in the USA, Europe and Japan for RAI-refractory DTC.

Sorafenib and lenvatinib both extended progression-free survival (PFS) compared with placebo in randomized phase III studies (Brose *et al.* 2014, Schlumberger *et al.* 2015). In the DECISION phase III trial, sorafenib extended median PFS to 10.8 months compared with 5.8 months in placebo-treated subjects (Brose *et al.* 2014). The SELECT phase III trial showed that the median PFS in lenvatinib-treated patients was 18.3 months versus 3.6 months for the placebo-treated group (Schlumberger *et al.* 2015). Overall survival (OS) did not differ from placebo in either study, most likely due to the crossover effect (whereby disease progression in patients receiving placebo enabled crossover to the study drug). Additionally, these MKIs were associated with various adverse events (AEs), such as hand–foot syndrome, rash, and diarrhea with sorafenib; and hypertension, fatigue, and proteinuria with lenvatinib (Brose *et al.* 2014, Schlumberger *et al.* 2015, Ito *et al.* 2016). The escape phenomenon (the use or activation of ‘alternative proangiogenic pathways’ that enable an escape from VEGF–VEGFR signaling) and treatment resistance are other common limitations of MKIs (Naoum *et al.* 2018). Hence, more effective and tolerable treatment options are still needed for these patients.

Vandetanib is an MKI that targets VEGFR 1–3, RET, and epidermal growth factor receptor (EGFR) (Wedge *et al.* 2002, Leboulleux *et al.* 2012). The randomized phase II ZACTHYF study in DTC showed a significant PFS advantage in patients with locally advanced or metastatic RAI-refractory disease treated with vandetanib compared with placebo (median PFS 11.1 vs 5.9 months, respectively; hazard ratio (HR) 0.63; 95% CI 0.43–0.92; *P* = 0.017) (Leboulleux *et al.* 2012). The VERIFY trial was designed to evaluate vandetanib in a larger population of tyrosine kinase inhibitor-naive patients with RAI-refractory DTC. VERIFY is a randomized, double-blind, placebo-controlled, multicenter phase III study to assess the efficacy and safety of 300 mg of vandetanib (Caprelsa; SAR390530 (formerly AstraZeneca ZD6474)) in patients with locally advanced or metastatic DTC that is refractory to RAI therapy and with documented progression.

## Materials and methods

### Patients

The patients were recruited from 66 sites across 12 countries in the USA, Europe, and Asia between 2013 and 2015. Patients eligible for enrollment were aged 18 years or older, provided informed consent, and had a confirmed histological diagnosis of locally advanced or metastatic DTC (including poorly differentiated thyroid cancer and follicular DTC) not amenable to surgical resection, external beam radiotherapy, or other local therapies. Measurable disease, as defined by Response Evaluation Criteria in Solid Tumors (RECIST) version 1.1 (Eisenhauer *et al.,* 2009), was required for eligibility. Measurable lesions with calcifications were not assessed as target lesions unless no other measurable lesion was available. Patients must have had disease progression (according to RECIST v1.1) within 14 months of randomization and be RAI-refractory/resistant, as defined by the following criteria: (i) one or more measurable lesions that do not demonstrate RAI uptake on a post-RAI scan performed under conditions of a low-iodine diet and adequate TSH elevation or recombinant human TSH stimulation; (ii) radiologically documented disease progression within 14 months of RAI therapy despite demonstration of radioactive avidity at the time of that treatment by pre- or post-treatment scanning; (iii) or disease progression despite having received the maximum lifetime activity of RAI (cumulative activity of RAI >600 mCi (22 GBq)). Other key inclusion criteria were TSH suppression below 0.5 mU/L, World Health Organization (WHO) or Eastern Cooperative Oncology Group (ECOG) performance status (PS) 0–2, and negative pregnancy test (urine or serum) for female patients of childbearing potential. The full list of inclusion and exclusion criteria is available in Supplementary Data (see section on supplementary materials given at the end of this article).

### Study design

Patients were randomized in a 1:1 ratio to receive vandetanib at a starting dose of 300 mg/day or placebo until disease progression. Once informed consent was obtained and patient eligibility confirmed, each patient was assigned a unique enrollment number and randomization code (patient number) via an ‘Interactive Voice Response System/Interactive Web Response System’ (IVRS/IWRS). After a 4-week screening period, patients still fulfilling all eligibility criteria at their next clinic visit were randomly assigned to study treatment using IVRS/IWRS. Randomization codes were assigned strictly sequentially by IVRS/IWRS as patients became eligible for randomization. If a patient withdrew from the study after enrollment or after receiving study treatment, they were not permitted to re-enter the study. The study drug was labeled using a unique MedID number, which was linked to the randomization scheme. Patients received the study drug or placebo dispensed as three 100 mg tablets, which were taken whole or dispersed in water, without crushing. The active and placebo tablets were identical and presented in the same packaging to ensure the blinding of the study treatment. During the initial 24-week treatment period, patient visits frequency occurred every 3 weeks. Thereafter, patient visit frequency changed to every 12 weeks. A 60-day follow-up visit was carried out upon intervention discontinuation.

**Figure 1:**
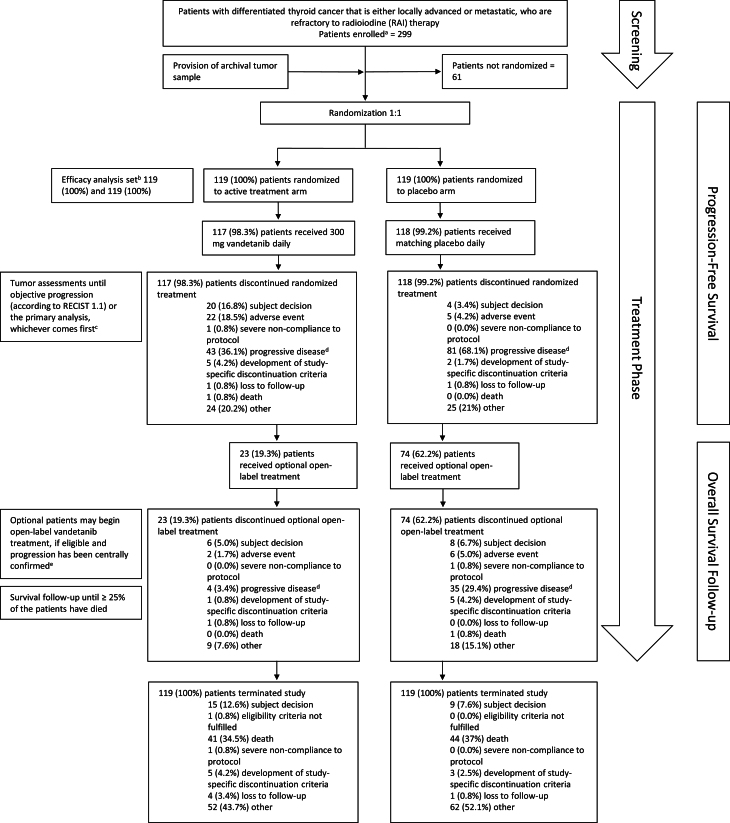
Study design and patient disposition. ^a^Informed consent received. ^b^All randomized patients, regardless of whether they took study treatment. ^c^Patients continued to receive randomized treatment until objective disease progression (RECIST version 1.1) or for as long as they received clinical benefit in the opinion of the investigator, unless any of the criteria for treatment discontinuation were met first. Patients who discontinued study treatment for reasons other than disease progression had to continue tumor assessments as per the Study Plan and until progression. ^d^The corresponding category in the electronic case report form was ‘Condition under investigation worsened’. ^e^Once PFS analysis had been performed, there was no longer a requirement for central confirmation of progression prior to open-label treatment. PFS, progression-free survival; RECIST, Response Evaluation Criteria in Solid Tumors.

At the end of the treatment period, upon centrally confirmed disease progression, patients were unblinded and offered open-label vandetanib, regardless of treatment arm, if deemed beneficial by the investigator (Fig. 1). Patients were followed up for survival every 12 weeks.

### Criteria for outcomes

PFS was defined as the time (in months) from randomization until the date of first documented disease progression or death (from any cause), whichever came first. Disease progression, as determined by RECIST v1.1, was defined as an at least 20% increase and an absolute increase of 5 mm in the sum of the longest diameter (LD) of target lesions, taking as reference the smallest sum LD recorded since the treatment started or the appearance of one or more new lesions. Disease progression was confirmed by a blinded independent central review. Analysis was performed by the Kaplan–Meier method. PFS was assessed every 12 weeks (up to 22 months).

Best objective response (BOR), objective response rate (ORR), and change in tumor size (TS) were assessed by RECIST measurements and taken every 12 weeks from randomization. TS was assessed at screening and then every 12 weeks, as well as at the discontinuation visit at 25.5 months. TS was evaluated once at least 155 progression events had occurred. OS was evaluated both at the time of the analysis of PFS (primary analysis, when approximately 155 progression events had occurred) and once at least 155 progression events had occurred and when 25% of patients had died of any cause. Following a preliminary review of primary efficacy data, *ad hoc* analyses were performed to evaluate the effect of TSH levels on PFS.

Safety and tolerability of vandetanib treatment were assessed by AEs (according to Common Terminology Criteria for Adverse Events (CTCAE) version 4) (US Department of Health and Human Services 2009), vital signs, laboratory parameters, and electrocardiography. Safety assessments were performed at baseline, weeks 1, 2, 4, 8, and 12, and then every 12 weeks thereafter until at least 155 progression events had occurred.

### Statistical considerations

This study was designed to test the null hypothesis (the true HR is 1; a 0% prolongation in PFS) vs the alternative hypothesis (the true HR is 0.63; a 59% prolongation in PFS for patients taking vandetanib) with >80% power and a two-sided 5% significance level. A minimum of 155 progression events were required. Assuming a median PFS of 6 months for placebo, a recruitment period of 18 months, a minimum follow-up of approximately 7.5 months, and 1:1 randomization, a minimum of 227 patients were to be randomized. This equates to a 3.5-month improvement in median PFS, or a median PFS of 9.5 months in the vandetanib treatment arm. The threshold value for the HR that would achieve *P* < 0.05 was 0.73 (median PFS of 8.2 months on the vandetanib-treated arm vs 6 months on the placebo arm).

Analysis of PFS was performed using a log-rank test (unadjusted model with treatment factor only). The comparison of treatments was estimated using the HR along with the corresponding two-sided 95% CI and two-sided *P* value. A Kaplan–Meier plot for PFS is presented with and without two-sided 95% CIs. Median PFS (with two-sided 95% CI) and the proportion of patients who were progression-free at 6 months, 1 year, and 2 years are presented by randomized treatment group. A log transformation was applied in the calculation of 95% CIs for median PFS. Sensitivity analyses of PFS were performed using PFS derived according to the central assessment of RECIST by the Core Imaging Laboratory. If the patient has no evaluable visits or does not have baseline data, they were censored at 0 days (day 1) unless they die within two visits (28 weeks) of baseline, when they would be deemed to progress at the date of death.

Additional information on statistical considerations can be found as part of Supplementary Data (Supplementary Statistical Considerations).

### Ethical approval

The protocol (legacy study number: D4203C00011, Sanofi study number: LPS14813) and its four amendments were submitted to independent Ethics Committees and/or Institutional Review Boards for review and written approval. The protocol complied with recommendations of the 18th World Health Congress (Helsinki, 1964), and all applicable amendments. The protocol also complied with the laws and regulations, as well as any applicable guidelines, of the countries where the study was conducted. The UPenn Institutional Review Board in Philadelphia, Pennsylvania, USA, and many other Ethics Committees and Institutional Review Boards situated in the involved countries approved the study.

## Results

### Patients

In total, 238 patients were randomly assigned to receive vandetanib (119 patients) or placebo (119 patients) (Fig. 1). Of these, 117 patients received vandetanib 300 mg, and 118 patients received placebo; three patients (1.3%) did not receive randomized treatment since two of them decided to withdraw from the study, and the remaining one no longer met the eligibility criteria (Supplementary Table 1). The baseline demographic and clinical characteristics of the vandetanib 300 mg and placebo groups were well-balanced (Table 1). Of the study population, 98.3% (*n* = 234) of patients had metastases (Table 1).

**Table 1:** Patient demographics and baseline clinical characteristics. For countries where only the year and month of birth are collected, the day of birth is imputed as the first day of the month. *BRAF* mutation status = *BRAF* VAL600GLU mutation status.

	**Vandetanib 300 mg(*n*=119)**	Placebo(*n*=119)
Age, years		
Mean (s.d.)	64.2 (9.69)	63.2 (11.02)
Age group, *n* (%)		
18 to <45 years	5 (4.2)	6 (5.0)
45 to <65 years	49 (41.2)	57 (47.9)
≥65 years	65 (54.6)	56 (47.1)
Sex, *n* (%)		
Male	49 (41.2)	55 (46.2)
Female	70 (58.8)	64 (53.8)
Race, *n* (%)		
White	87 (73.1)	80 (67.8)
Black or African American	0 (0.0)	3 (2.5)
Asian	31 (26.1)	31 (26.3)
Other	1 (0.8)	4 (3.4)
Histology type, *n* (%)		
Papillary	69 (58.0)	71 (59.7)
Follicular^a^	29 (24.4)	28 (23.5)
Other	19 (16.0)	20 (16.8)
Missing	2 (1.7)	0 (0.0)
Time from diagnosis to randomization, months	*n* = 118	*n* = 119
Median (minimum, maximum)	76.07 (0.2, 381.9)	61.90 (4.8, 345.3)
Overall disease classification, *n* (%)		
Metastatic^b^	117 (98.3)	117 (98.3)
Locally advanced^c^	1 (0.8)	2 (1.7)
Missing	1 (0.8)	0 (0.0)
*BRAF* mutation status		
Mutated	36 (30.3)	39 (32.8)
Wild type	59 (49.6)	58 (48.7)
Unknown/missing	24 (20.2)	22 (18.5)
RET/PTC1 fusion		
Detected	5 (4.2)	7 (5.9)
Not detected	70 (58.8)	74 (62.2)
Unknown/missing	44 (37.0)	38 (31.9)
RET/PTC3 fusion		
Detected	1 (0.8)	0
Not detected	74 (62.2)	81 (68.1)
Unknown/missing	44 (37.0)	38 (31.9)
Combined RET/PTC1 and RET/PTC3 response		
Detected	6 (5.0)	7 (5.9)
Not detected	69 (58.0)	74 (62.2)
Unknown/missing	44 (37.0)	38 (31.9)
Patients with previous external beam radiation therapy, *n* (%)	51 (42.9)	43 (36.1)
Metastasis at lung site		
Yes	109 (91.6)	114 (95.8)
No	10 (8.4)	5 (4.2)

^a^Follicular includes rarer variants of thyroid cancer of follicular cell origin, such as oncocytic carcinoma and poorly differentiated thyroid cancer; ^b^Metastatic = the patient has any distant metastatic sites of disease; ^c^Locally advanced = the patient has only locally advanced sites of disease.

PTC, papillary thyroid carcinoma; RET, rearranged during transfection.

### Efficacy

**Figure 2:**
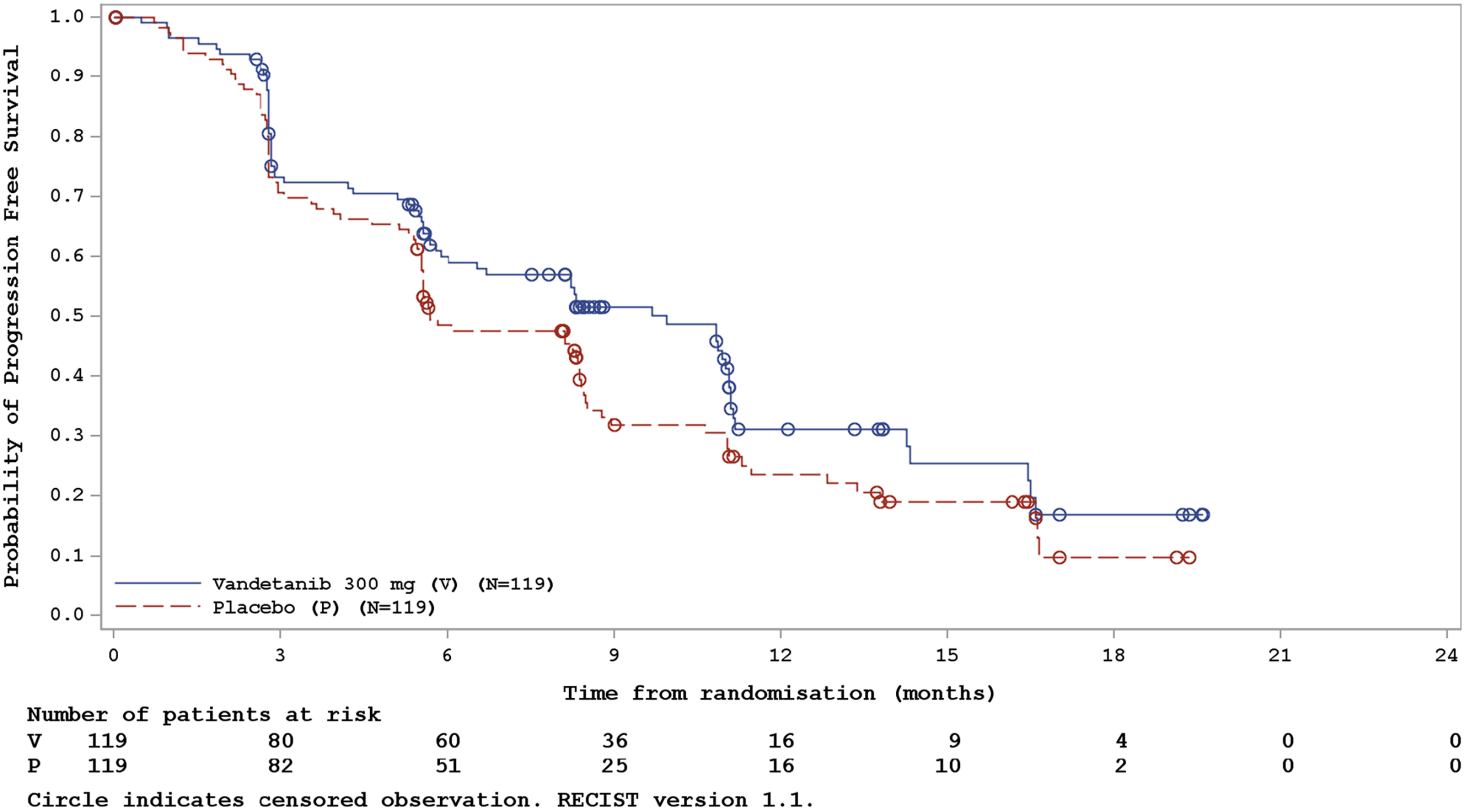
Kaplan–Meier curve of progression-free survival (efficacy analysis set).

**Figure 3:**
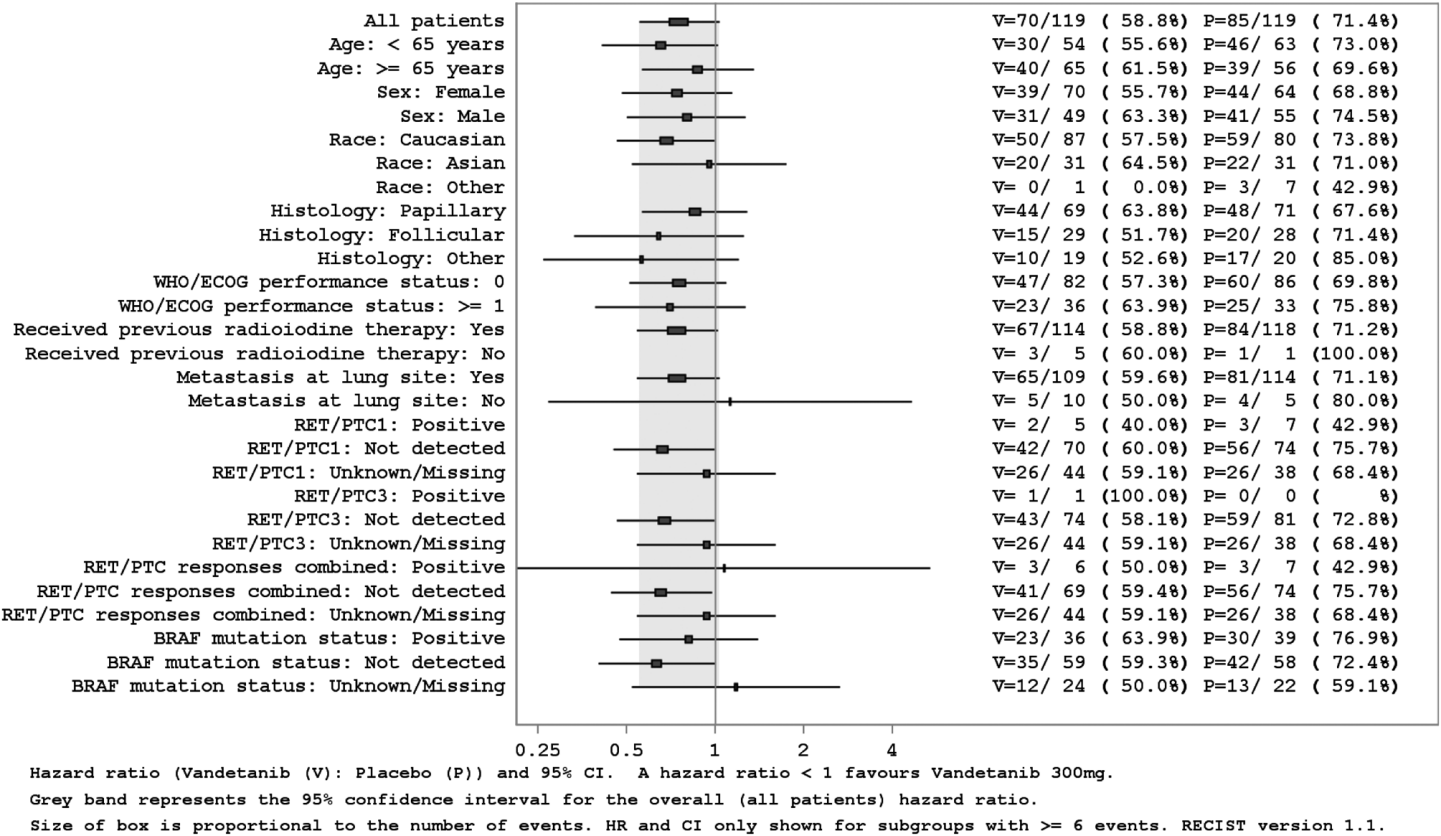
Forest plot of progression-free survival by subgroup (efficacy analysis set). Hazard ratio (HR) and 95% CI. HR < 1 favors vandetanib 300 mg over placebo. Gray band represents the 95% CI for the overall (all patients) HR. Size of box is proportional to the number of events. HR and CI shown only for subgroups with ≥6 events. RECIST version 1.1. ECOG, Eastern Cooperative Oncology Group; PTC, papillary thyroid carcinoma; RECIST, Response Evaluation Criteria in Solid Tumors; RET, rearranged during transfection; WHO, World Health Organization.

Median PFS was 10.0 months in the vandetanib group and 5.7 months in the placebo arm (Table 2 and Fig. 2). PFS in patients treated with vandetanib was not significantly improved compared with placebo-treated patients, as assessed by both local review (HR: 0.75; 95% CI: 0.55–1.03; *P* = 0.080; Supplementary Table 2) and independent central review (HR: 0.75; 95% CI: 0.55–1.02; *P* = 0.065; Supplementary Table 2). Subset analysis demonstrated that PFS was favorable (although not statistically significant) in the vandetanib-treated vs the placebo arm for all but three listed groups of patients (no metastases at lung site; RET/PTC1 and RET/PTC3 (combined) detected; *BRAF* mutation status unknown/missing), as shown in the forest plot (Fig. 3).

**Table 2:** Summary of efficacy in patients with differentiated thyroid cancer. Patients who did not have measurable disease at baseline are excluded from this table and the denominator in accordance with RECIST version 1.1 criteria.

	Vandetanib 300 mg	Placebo
PFS^a^	*n* = 119	*n* = 119
Total number of events^b^	70	85
Median PFS,^c^ months (95% CI)	10.0 (6.0–11.1)	5.7 (5.5–8.4)
Progression-free at 6 months, % (95% CI)	60.0 (50.1–68.5)	48.5 (39.1–57.3)
Progression-free at 1 year, % (95% CI)	31.1 (21.0–41.7)	23.6 (15.3–33.0)
Progression-free at 18 months, % (95% CI)	16.9 (7.9–28.9)	9.8 (3.3–20.6)
Best objective response	*n* = 119	*n* = 118
Response, *n* (%)		
Total	6 (5.0)	0 (0.0)
Complete response^d^	0 (0.0)	0 (0.0)
Partial response^d^	6 (5.0)	0 (0.0)
Non-response, *n* (%)		
Total	113 (95.0)	118 (100.0)
Stable disease ≥12 weeks	74 (62.2)	76 (64.4)
Progression	34 (28.6)	39 (33.1)
RECIST progression	31 (26.1)	33 (28.0)
Death	3 (2.5)	6 (5.1)
Not evaluable	5 (4.2)	3 (2.5)
Stable disease <12 weeks	1 (0.8)	0 (0.0)
Incomplete post-baseline assessment^e^	4 (3.4)	3 (2.5)

^a^Progression includes death in the absence of RECIST progression; ^b^Progression/death events that do not occur within 28 weeks of the last evaluable RECIST assessment (or randomization in the absence of an evaluable baseline RECIST assessment) are censored and therefore excluded in the number of events; ^c^Calculated using the Kaplan–Meier technique; ^d^Response does not require confirmation; ^e^This also includes patients with disease progression or who died but had insufficient tumor assessment data prior to the progression/death.

PFS, progression-free survival; RECIST, Response Evaluation Criteria in Solid Tumors.

The log-rank unadjusted test showed that vandetanib treatment was associated with a slight decrease in the risk of mortality, and modest increased chances of PFS were observed in patients treated with vandetanib who belong to the following subgroups: <65 years of age, had follicular histology, and for whom RET/PTC1, RET/PTC3, and BRAF mutation were not detected (Supplementary Subgroup Analysis).

**Figure 4:**
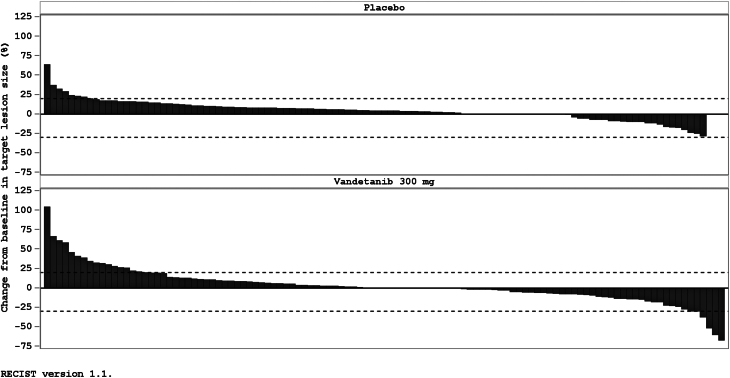
Waterfall plot of best percentage change in target lesion size from baseline (efficacy analysis set).

**Figure 5:**
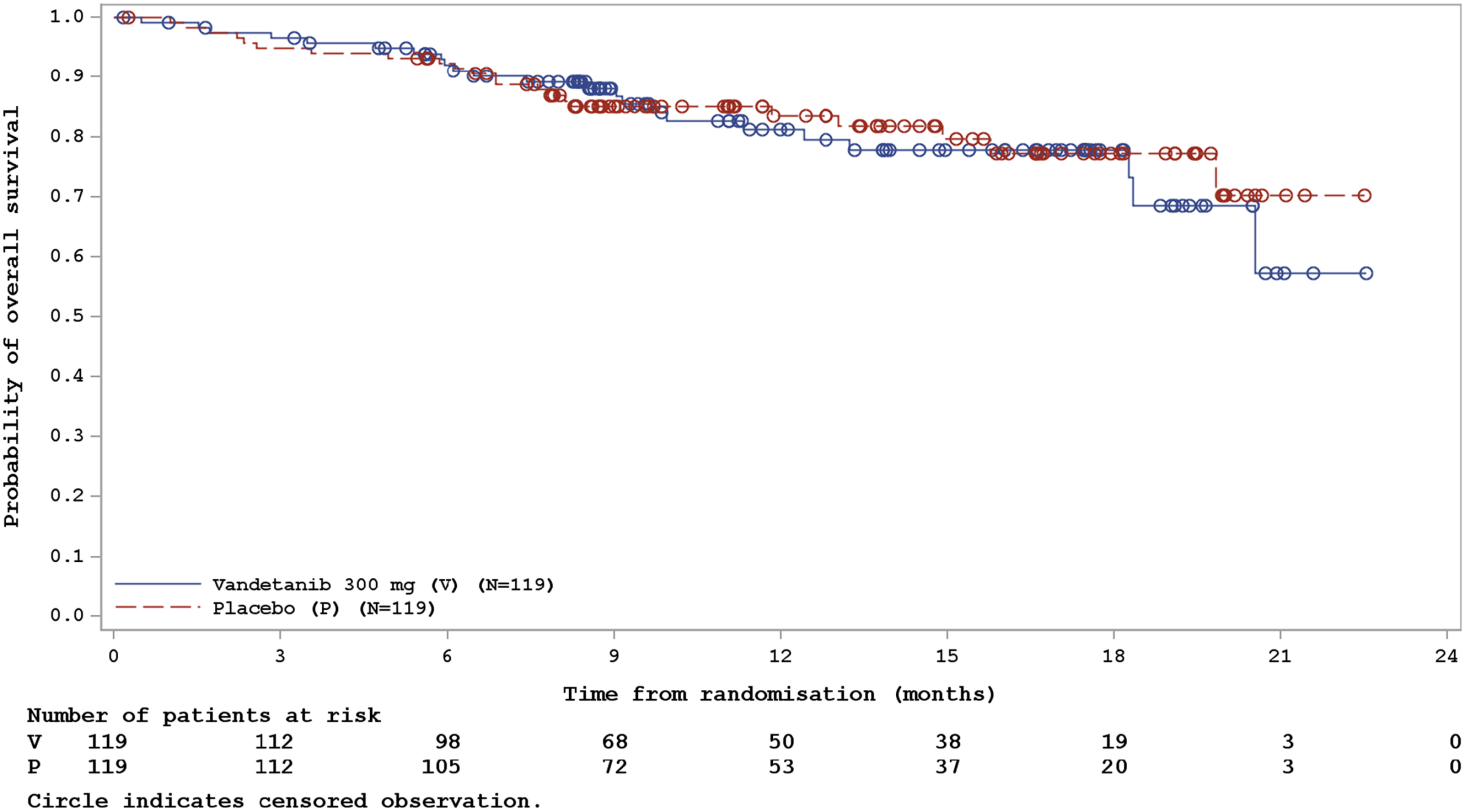
Kaplan–Meier curve for overall survival (efficacy analysis set). Circle indicates censored observation.

A partial response was observed in six of 119 (5.0%) patients in the vandetanib arm. None of the 118 patients in the placebo arm responded (Table 2). Due to the small number of responders, it was not meaningful to calculate the ORR. The proportion of patients with a reduction in target lesion size was greater in the vandetanib-treated group than in the placebo-treated group (Fig. 4). The Kaplan–Meier plot of OS data showed no difference between treatment arms (*P* = 0.956) (Fig. 5).

### Exploratory analysis – thyroid-stimulating hormone levels

Mean TSH and median PFS were determined in 113 and 116 patients who received vandetanib 300 mg and placebo, respectively. Of the study group, 76.1% of patients experienced an increase in serum TSH within 12 weeks of beginning vandetanib (Supplementary Fig. 1). In many but not all cases, this increase was ≥100% and prompted adjustment of the patient’s levothyroxine dose, such that 59 patients in the vandetanib arm had mean TSH levels below 0.5 mU/L, and 37 had mean TSH levels below 0.1 mU/L during the randomized treatment period (Table 3).

**Table 3:** Mean thyroid-stimulating hormone and median progression-free survival (efficacy analysis set). *n* is the number of patients in the respective subgroup with a baseline and at least one post-baseline TSH value in the randomized treatment period. Patients without baseline or post-baseline TSH are excluded from this table.

	*n*	Mean TSH^a,b^(mU/L)	Mean PFS^c^(months)
Vandetanib 300 mg			
All	113	1.48	10.8
Mean TSH <0.5 mU/L^a^	59	0.12	10.9
Mean TSH <0.1 mU/L^a^	37	0.04	11.1
Placebo			
All	116	0.34	5.8
Mean TSH <0.5 mU/L^a^	103	0.10	5.7
Mean TSH <0.1 mU/L^a^	69	0.03	8.1

^a^Mean TSH per patient is computed from the randomized treatment period, including the baseline value; ^b^The average across patients is shown; ^c^Calculated using the Kaplan–Meier technique.

PFS, progression-free survival; TSH, thyroid-stimulating hormone.

For patients with mean TSH levels below 0.5 mU/L during the randomized treatment period, median PFS was 10.9 months for vandetanib and 5.7 months for placebo (Table 3), whereas in patients with mean TSH levels below 0.1 mU/L during the randomized treatment period, median PFS was 11.1 months for vandetanib (*n* = 37) and 8.1 months for placebo (*n* = 69; Table 3). In a Cox proportional hazards model, adjusting for the most recent TSH above 0.1 mU/L prior to disease progression or death (a low value was a surrogate indicator that TSH control had been maintained or regained), the HR for vandetanib compared with placebo was 0.62 (95% CI: 0.44─0.87; *P* = 0.005; Supplementary Table 2). The absolute TSH values showed more variability in the vandetanib-treated group than in the placebo-treated group (Supplementary Fig. 1).

### Safety and tolerability

Of the 235 patients on randomized treatment, 97.4% of patients in the vandetanib arm and 89.8% in the placebo arm experienced at least one AE (Table 4). More patients in the vandetanib-treated group had discontinued the study treatment due to AEs than in the placebo group (18.8% vs 4.2%, respectively; Table 4). The most frequently reported AEs in the vandetanib-treated group are shown in Table 5. Thirty-five (29.9%) patients receiving vandetanib and 20 (16.9%) patients receiving placebo reported serious AEs (SAEs). The most frequently reported SAEs in the vandetanib-treated group were pneumonia and diarrhea. More deaths were reported in the vandetanib-treated group (33 patients; 28.2%) than in the placebo group (16 patients; 13.6%) (Table 4). Most of the deaths were related to disease under investigation (24 patients (20.5%) in the vandetanib-treated group and 10 patients (8.5%) in the placebo group; Table 4). Three deaths were considered related to the study treatment: one due to a lung abscess in the vandetanib arm and two due to acute myocardial infarction and dermatitis bullous in the placebo arm. There were eight (6.8%) patients in the vandetanib arm and five (4.2%) in the placebo arm who experienced an SAE leading to death (Table 6).

**Table 4:** Summary of adverse events, serious adverse events, relative dose intensity, and deaths while on randomized treatment. Includes AEs with an onset date on or after the date of the first randomized treatment and up to and including 60 days following the date of the last dose if on randomized treatment, excluding AEs with onset on or after the date of the first open-label treatment; includes two AEs with missing start dates; death related to disease under investigation is determined by the investigator; rows are mutually exclusive, patients are only reported in one category; deaths on or after the date of the first open-label vandetanib are excluded from this table.

	Vandetanib 300 mg(*n*= 117)	Placebo(*n = *118)
AEs and SAEs, *n* (%)		
Any AE^a^	114 (97.4)	106 (89.8)
Causally related to study treatment^b^	110 (94.0)	56 (47.5)
Leading to dose reduction of study treatment	35 (29.9)	4 (3.4)
Leading to dose interruption of study treatment	32 (27.4)	11 (9.3)
Any AE of CTCAE grade *≥*3	65 (55.6)	30 (25.4)
Causally related to study treatment^b^	44 (37.6)	7 (5.9)
Any SAE	35 (29.9)	20 (16.9)
Causally related to study treatment^b^	17 (14.5)	3 (2.5)
Any AE leading to discontinuation of study treatment	22 (18.8)	5 (4.2)
Causally related to study treatment^b^	14 (12.0)	1 (0.8)
RDI^c^	*n* = 116	*n* = 118
Mean (s.d.)	88.5 (19.47)	98.7 (5.92)
Median (minimum, maximum)	100.0 (33, 100)	100.0 (61, 100)
25th, 75th percentile	79, 100	100, 100
Deaths, *n* (%)		
Total	33 (28.2)	16 (13.6)
Death related to disease under investigation only	24 (20.5)	10 (8.5)
AE with outcome of death only (within 60 days after last randomized drug)	5 (4.3)	4 (3.4)
AE with outcome of death only (>60 days after last randomized drug)	0 (0.0)	0 (0.0)
Patients with death related to disease and an AE with outcome of death	3 (2.6)	0 (0.0)
Other deaths^d^	1 (0.9)	2 (1.7)

^a^Patients with multiple events in the same category are counted only once in that category. Patients with events in more than one category are counted once in each of those categories; ^b^As assessed by the investigator; ^c^RDI is the actual cumulative dose as a percentage of the intended cumulative dose delivered up to the earlier of progression (or a censoring event) or the actual last day of dosing of randomized treatment; ^d^Patients who died and are not captured in the earlier categories.

AE, adverse event; CTCAE, Common Terminology Criteria for Adverse Events; PFS, progression-free survival; RDI, relative dose intensity; SAE, serious adverse event.

**Table 5:** Most common adverse events while on randomized treatment (safety analysis set). The most common is defined as a total frequency of >10% (in any treatment group). Includes AEs with an onset date on or after the date of the first randomized treatment and up to and including 60 days following the date of the last dose of randomized treatment, excluding AEs with onset on or after the date of the first open-label treatment.

	Vandetanib 300 mg(*n*= 117)		Placebo(*n = *118)	
	Any grade	CTCAE grade ≥ 3	Any grade	CTCAE grade ≥ 3
Patients with any AE,^a^*n* (%)	114 (97.4)	65 (55.6)	106 (89.8)	30 (25.4)
Diarrhea	80 (68.4)	9 (7.7)	26 (22.0)	3 (2.5)
Hypertension	48 (41.0)	19 (16.2)	8 (6.8)	0
Rash	37 (31.6)	3 (2.6)	6 (5.1)	0
Electrocardiogram QT prolonged	36 (30.8)	12 (10.3)	4 (3.4)	1 (0.8)
Nausea	22 (18.8)	0	17 (14.4)	1 (0.8)
Asthenia	21 (17.9)	1 (0.9)	15 (12.7)	1 (0.8)
Fatigue	13 (11.1)	1 (0.9)	13 (11.0)	2 (1.7)
Headache	16 (13.7)	0	10 (8.5)	0
Decreased appetite	22 (18.8)	2 (1.7)	2 (1.7)	1 (0.8)
Dry skin	18 (15.4)	0	6 (5.1)	0
Cough	11 (9.4)	0	12 (10.2)	0
Hypocalcemia	18 (15.4)	4 (3.4)	3 (2.5)	0
Vomiting	9 (7.7)	0	12 (10.2)	1 (0.8)
Alanine aminotransferase increased	16 (13.7)	2 (1.7)	4 (3.4)	1 (0.8)
Back pain	8 (6.8)	0	12 (10.2)	1 (0.0)
Dyspnea	7 (6.0)	3 (2.6)	13 (11.0)	4 (3.4)
Aspartate aminotransferase increased	15 (12.8)	2 (1.7)	4 (3.4)	1 (0.8)
Insomnia	15 (12.8)	1 (0.9)	2 (1.7)	0
Dermatitis acneiform	13 (11.1)	0	2 (1.7)	0
Photosensitivity reaction	15 (12.8)	3 (2.6)	0	0
Urinary tract infection	12 (10.3)	1 (0.9)	3 (2.5)	0

^a^AEs listed in decreasing frequency of preferred terms (in the total study sample).

AE, adverse event.

**Table 6:** Serious adverse events with the outcome of death (safety analysis set). Patients with multiple AEs with an outcome of death are counted once for each system organ class/preferred term. Includes AEs with an onset date on or after the date of the first randomized treatment and up to and including 60 days following the date of the last dose of randomized treatment, excluding AEs with onset on or after the date of the first open-label treatment.

	Vandetanib 300 mg (*n*= 117)	Placebo(*n*= 118)
Patients with any AE with the outcome of death, *n* (%)	8 (6.8)	5 (4.2)
Infections and infestations	4 (3.4)	2 (1.7)
Lung abscess	1 (0.9)	0
Lung infection	1 (0.9)	0
Pneumonia	2 (1.7)	2 (1.7)
Cardiac disorders	2 (1.7)	2 (1.7)
Acute myocardial infarction	0	1 (0.8)
Cardiac arrest	1 (0.9)	0
Cardiac failure	0	1 (0.8)
Cardiac failure acute	1 (0.9)	0
Respiratory, thoracic, and mediastinal disorders	4 (3.4)	2 (1.7)
Acute respiratory failure	1 (0.9)	1 (0.8)
Dyspnea	1 (0.9)	0
Pneumonia aspiration	1 (0.9)	0
Pneumothorax	1 (0.9)	0
Respiratory failure	0	1 (0.8)
Renal and urinary disorders	1 (0.9)	0
Acute kidney injury	1 (0.9)	0
General disorders and administration-site conditions	1 (0.9)	0
Multiple organ dysfunction syndrome	1 (0.9)	0

AE, adverse event.

## Discussion

VERIFY investigated the efficacy and safety of vandetanib in patients with locally advanced or metastatic RAI-refractory DTC but did not confirm the findings of the previous phase II ZACTHYF study that showed a significant PFS advantage in patients treated with vandetanib compared with placebo: median PFS 11.1 vs 5.9 months, respectively (Leboulleux *et al.* 2012).

In the DECISION study, sorafenib demonstrated improvements in PFS compared with placebo, similar to the results of the present study: median PFS of 10.8 months in the sorafenib arm and 5.8 months in the placebo arm, with data that were statistically significant (Brose *et al.* 2014). Similarly, the SELECT study also showed a meaningful difference in PFS with lenvatinib compared with placebo (Schlumberger *et al.* 2015). However, it is worth considering that the patient population enrolled in the current trial was smaller than in either of the other two (active treatment group of 119 patients versus 207 and 261 patients, respectively). The sample size, together with other statistical assumptions, may have contributed to the lack of a statistically significant difference in the primary endpoint in this trial. In the subgroup analysis of the primary endpoint, it was notable that the only three subgroups in which PFS was not favorable in the vandetanib vs the placebo arm featured particularly small sample sizes (6, 10, and 24 patients).

Other differences in clinically related factors might have also contributed to the lack of statistical significance. With the use of an MKI (vandetanib), serum TSH levels commonly increase and might contribute to DTC tumor growth (Haymart *et al.* 2008, Kim *et al.* 2013, Ozemir *et al.* 2015). Therefore, TSH level monitoring is recommended, with modification of the levothyroxine dose where indicated to reach suppressed TSH levels in accordance with the American Thyroid Association (ATA) guidelines (<0.1 mU/L) (Haugen *et al.* 2016).

It is worth noting that serum TSH < 0.5 mIU/L was among the inclusion criteria for both the sorafenib and lenvatinib trials (Brose *et al.* 2014, Schlumberger *et al.* 2015), as was the case here, but the TSH level observed in 93 of 117 patients in the vandetanib arm during the treatment period exceeded the ATA-recommended level of <0.1 mU/L (Haugen *et al.* 2016). Future studies of vandetanib – and indeed other kinase inhibitors – in the RAI-refractory DTC population should require the adequate control of TSH levels during the study treatment period to ensure the generation of robust data.

The safety observations were consistent with anticipated effects of vandetanib and were in line with other drugs of the same class. More AEs and SAEs were reported in the vandetanib-treated group of patients compared to the placebo during randomized treatment, which is consistent with observations in the placebo-controlled trials of sorafenib and lenvatinib (Brose *et al.* 2014, Schlumberger *et al.* 2015). The occurrence and severity of AEs or SAEs can lead to the inability of patients to go back on treatment or result in delays in treatment. Therefore, early intervention when AEs occur may improve adherence and might lead to improved responses (i.e. more patients staying on treatment longer).

The most frequently observed AEs included diarrhea in 68.4% of patients treated with vandetanib vs 22% of patients in the placebo arm, hypertension in 41.8% vs 6.8%, rash (any grade) in 31.6% vs 5.1%, and QT prolongation in 30.8% vs 3.4% (Table 5). These observations are consistent with the AEs reported in previous vandetanib clinical trials (Leboulleux *et al.* 2012).

Among gastrointestinal AEs, vandetanib-arm patients reported higher rates of nausea (18.8% vs 14.4%), decreased appetite (18.8% vs 1.7%), and lower rates of vomiting (7.7% vs 10.2%) compared to the placebo arm (Table 5). Due to the QT interval risk, 5-HT3 antagonists and high-dose metoclopramide should be avoided. Palonosetron, aprepitant, a neurokinin 1 receptor antagonist, and peristaltic retarders or mild opioids like codeine may be considered for the management of gastrointestinal AEs (Grande *et al.* 2013).

Cardiovascular AEs include hypertension. Close monitoring of blood pressure in the first months of treatment is recommended, and initiation of treatment with angiotensin-converting enzyme inhibitors, calcium antagonists, or beta-blockers should be initiated if needed (Grande *et al.* 2013).

Similarly to rash, other dermatological AEs occurred more frequently in patients in the vandetanib arm than in those in the placebo-arm and involve dry skin (15.4% vs 5.1%), dermatitis acneiform (11.1% vs 1.7%), and photosensitivity reactions (12.8% vs 0%) (Table 5). It is recommended to manage skin reactions with photoprotection, avoidance of drying agents, and patient education (Grande *et al.* 2013).

QT prolongation usually occurs within the initial 3 months (Grande *et al.* 2013). It is recommended to discontinue vandetanib at QT ≥ 500 ms, to resume it at a lower dose upon QT normalization, to monitor electrolytes, to maintain TSH levels, and to avoid QT-prolonging drugs (Grande *et al.* 2013).

Despite there being no significant difference in OS between treatment groups in the current study (*P* = 0.956), a higher number of deaths was recorded in the active treatment group (28.2% vs 13.6% in the placebo arm in the current study), consistent with the findings of the sorafenib trial (12 (5.8%) vs 6 (2.9%), respectively), and may reflect, in part, the fact that patients are on treatment twice as long as they are on placebo (Brose *et al.* 2014).

In conclusion, VERIFY did not reach its primary endpoint. This result does not confirm the preliminary findings from the previous randomized phase II study, perhaps due to sample size or the potential effect of TSH on DTC growth (Haymart *et al.* 2008, Kim *et al.* 2013, Ozemir *et al.* 2015) during treatment with vandetanib. Furthermore, no new safety concerns were reported. In studies focusing on RAI-refractory DTC, it is crucial to carefully consider the statistical design and ensure that TSH is adequately maintained at the recommended level of less than 0.1 mU/L.

## Supplementary Materials

Supplementary Figure 1

Supplementary Data

SUPPLEMENTARY TABLE S1. PATIENT DEMOGRAPHICS OF SUBJECTS ENROLLED, BUT THAT DISCONTINUED THE STUDY BEFORE RECEIVING RANDOMIZED TREATMENT

SUPPLEMENTARY TABLE S2. SUMMARY OF EFFICACY IN PATIENTS WITH DIFFERENTIATED THYROID CANCER

## Declaration of interest

MSB has received honoraria for scientific consultancy (advisory roles) and trial funding from Sanofi, AstraZeneca, Bayer Healthcare, Loxo Oncology, Eisai, and Novartis. LB has received honoraria for scientific consultancy roles (speaker and advisory roles) from Novartis, Bristol-Myers Squibb, MSD, Roche, Ipsen, Bayer, Eisai, Amgen, and AstraZeneca. JB was an employee of Sanofi at the time of this analysis. JC has received honoraria for scientific consultancy roles (speaker and advisory roles) from Novartis, Pfizer, Ipsen, Exelixis, Bayer, Eisai, Advanced Accelerator Applications, Amgen, Sanofi, and Merck Serono, and research support from Eisai, Novartis, Ipsen, AstraZeneca, Pfizer, and Advanced Accelerator Applications. RMC is an employee of Sanofi. PC is an employee of Sanofi and a Sanofi stock shareholder. RE is a consultant for Eisai, Sanofi, Exelixis, and Loxo Oncology. DF-S has received honoraria for scientific consultancy roles (speaker and advisory roles) from Novartis, Pfizer, Ipsen, Bayer, Eisai, AstraZeneca, Amgen, Sanofi, and Merck Serono, and research support from Novartis, Ipsen, and Pfizer. SLe is a consultant for Eisai, Sanofi, Bayer, and Loxo Oncology. SLi is a Sanofi employee and Sanofi stock shareholder. IS has received honoraria for scientific consultancy roles (speaker and advisory roles) from Bayer and Eisai, and research support from Eisai. MHT received fees for consulting/advisory board participation for Bristol-Myers Squibb, Eisai Inc., Blueprint Medicines, Loxo Oncology, Bayer, Array Biopharma, Arqule, and Novartis, and unbranded speaking engagements with Bristol-Myers Squibb and Eisai Inc. ZW has no conflicts to disclose. LJW has received honoraria for scientific consultancy from Bayer, Eisai, Loxo Oncology, and Merck. FPW has received honoraria for scientific consultancy from Bayer, CUE Biopharmaceuticals, Fusion Pharmaceuticals, Eisai, Loxo Oncology, and Merck. MS has received consulting fees and research grants from Bayer, Eisai, Exelixis-IPSEN, and Sanofi. MS is on the editorial board of *Endocrine-Related Cancer*; MS was not involved in the review or editorial process for this paper, on which he is listed as an author.

## Funding

This study was sponsored by Sanofihttp://dx.doi.org/10.13039/100004339.

## Data availability

Restrictions apply to the availability of data generated or analyzed during this study to preserve patient confidentiality or because they were used under license. The corresponding author will, on request, detail the restrictions and any conditions under which access to some data may be provided.

## Author contribution statement

MSB, LB, JB, JC, RMC, PC, RE, DF-S, SLe, SLi, IS, MHT, ZW, LJW, FPW, and MS provided analysis and interpretation of the study data, critical revision of the draft content, and final approval of the submitted manuscript.

## Acknowledgements

We thank Alicja Bulsiewicz, PhD, and Jonathan Morton, PhD, from Comradis Ltd, who provided medical writing assistance funded by Sanofi.
