# Supplementary figures and images for: Vandetanib in locally advanced or metastatic differentiated thyroid cancer refractory to radioiodine therapy
Source: Endocr Relat Cancer. 2024 Jul 2;31(8):e230354. doi: 10.1530/ERC-23-0354 (PMC11301419; doi:10.1530/ERC-23-0354)

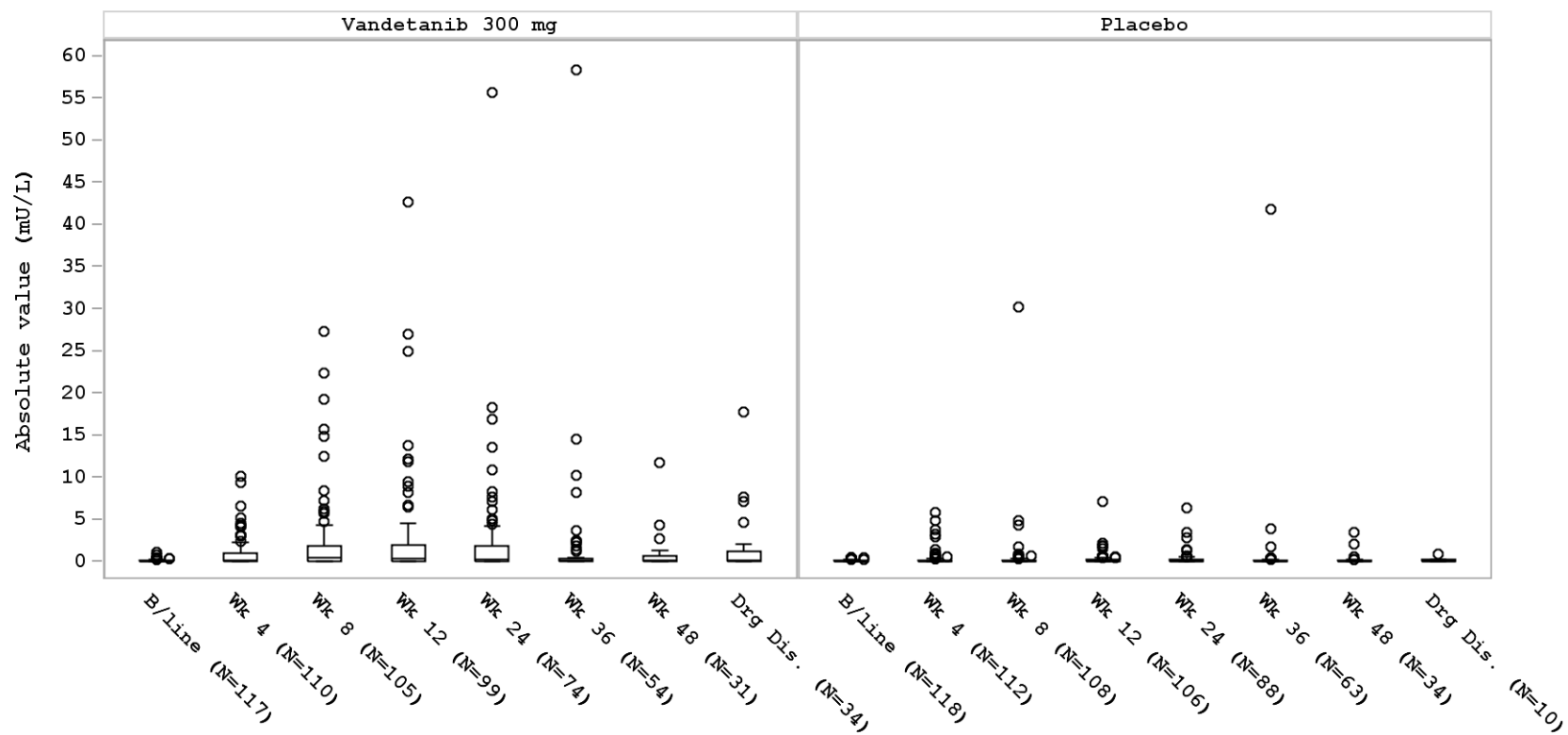

Supplement: Supplementary Figure 1 [file supplementary_figure_1.pdf]
