# Supplementary material for: Vandetanib in locally advanced or metastatic differentiated thyroid cancer refractory to radioiodine therapy
Source: Endocr Relat Cancer. 2024 Jul 2;31(8):e230354. doi: 10.1530/ERC-23-0354 (PMC11301419; doi:10.1530/ERC-23-0354)
Supplement: Supplementary Data [file supplementary_data.pdf]

Supplementary Data

Vandetanib in Locally Advanced or Metastatic Differentiated Thyroid Cancer Refractory to Radioiodine Therapy

Inclusion and exclusion criteria from amended protocol 04, legacy Study Code: D4203C00011 (Sanofi Study Code: LPS14813)

Inclusion criteria

For inclusion in the study patients must fulfil the following criteria:

1. Provision of informed consent prior to any study specific procedures.
2. Provision of informed consent to provide a sample of a previously obtained archival tumour biopsy (likely from diagnosis) for molecular analysis.
3. Female or male aged 18 years and older.
4. Previously confirmed histological diagnosis of locally advanced or metastatic differentiated (excluding minimally invasive follicular) thyroid cancer not amenable to surgical resection, external beam radiotherapy or other local therapies. Documentation must be provided in the patient's medical records.
5. Measurable disease defined as at least one lesion, not irradiated within 12 weeks of the date of randomisation, that can be accurately measured at baseline as ≥10 mm in the longest diameter (lymph nodes must have a short axis ≥15 mm) with CT or MRI and that is suitable for accurate repeated measurements. Measurable lesions with calcifications should not be assessed as target lesions unless no other measurable lesion is available.
6. Patients must have progressed within 14 months and be RAI-refractory/resistant or unsuitable for RAI as defined by one of the following:
    - One or more measurable lesions that do not demonstrate RAI uptake on a post-RAI scan performed under conditions of a low iodine diet and adequate TSH elevation or recombinant human TSH (rhTSH) stimulation.
    - Radiologically documented progression within 14 months of RAI therapy, despite demonstration of radioiodine avidity at the time of that treatment by pre- or post-treatment scanning.
    - Cumulative dose of RAI of > 600 mCi or 22 gigabequerels (GBq)
7. TSH suppression below 0.5 mU/L.
8. World Health Organisation (WHO) or Eastern Cooperative Oncology Group (ECOG) Performance status 0-2.
9. Negative pregnancy test (urine or serum) for female patients of childbearing potential.

Exclusion criteria

Patients should not enter the study if any of the following exclusion criteria are fulfilled:

1. Inadequate organ function as defined by:
    - Alanine aminotransferase (ALT), aspartate aminotransferase (AST), or alkaline phosphatase (ALP) greater than 2.5 x ULN, or greater than 5.0 x ULN if judged by the Investigator to be related to liver metastases.

- Serum bilirubin greater than 1.5 x ULN. This criterion does not apply to patients with known Gilbert's Disease.
- Creatinine clearance <50 ml/min (calculated by Cockcroft-Gault formula).

2. Risk of prolonged QTc as defined by:
    - Current concomitant therapy with any medications that are known to be associated with Torsades de Pointes or potent inducers of cytochrome P450 3A4 (CYP3A4).
    - History of QT prolongation associated with other medications that required discontinuation of that medication.
    - Congenital long QT syndrome.
    - QTcB correction unmeasurable or >480 ms on screening ECG.
        - If a patient has a QTcB interval >480 ms on screening ECG, the screening ECG may be repeated twice (at least 24 hours apart) for a total of 3 ECGs. The average QTcB from the three screening ECGs must be ≤480 ms in order for the patient to be eligible for the study.
3. The following electrolyte values are excluded:
    - Potassium <4.0 mmol/L despite supplementation, or above the CTCAE Grade 1 upper limit, at the time of randomisation.
    - Magnesium below the normal range despite supplementation, or above the CTCAE Grade 1 upper limit, at the time of randomisation.
    - Calcium (ionized or serum) below the normal range despite supplementation, or above the Grade 1 upper limit, at the time of randomisation. If serum calcium is used, correction should be applied to account for hypoalbuminemia, if present, where the corrected serum calcium (mg/dL) is equal to measured serum Ca (mg/dL) + 0.8 x (4 - serum albumin g/dL).
4. History of arrhythmia (multifocal premature ventricular contractions, bigeminy, trigeminy, ventricular tachycardia), which is symptomatic or requires treatment (CTCAE Grade 3), symptomatic or uncontrolled atrial fibrillation despite treatment, or asymptomatic sustained ventricular tachycardia. Patients with atrial fibrillation controlled by medication are permitted.
5. Significant cardiac event (e.g., myocardial infarction), superior vena cava syndrome, New York Heart Association (NYHA) classification of heart disease ≥2 within 12 weeks before randomisation, or presence of cardiac disease that in the opinion of the Investigator increases the risk of ventricular arrhythmia.
6. Previous or current malignancies of other histology within the last 5 years, with the exception of tumours associated with in situ carcinoma of the cervix, and adequately treated basal cell or squamous cell carcinoma of the skin.
7. Unstable brain metastases or spinal cord compression that requires treatment, unless the treatment ended at least 4 weeks before randomisation and the condition has been stable without steroid treatment for at least 10 days.
8. Any unresolved chronic toxicity greater than CTCAE Grade 2, except alopecia.
9. Major surgery (includes any surgery that carries significant risk of blood loss, extended periods of general anaesthesia, or requires at least an overnight hospital admission) within 28 days before randomisation.
10. Participation in a clinical study and/or receipt of an investigational drug within 28 days prior to randomisation, or last dose of prior chemotherapy within 28 days prior to randomisation (participation in the survival follow-up period of a study is not an exclusion criterion).

11. Previous exposure to vandetanib.
12. Previous enrolment or randomisation in the present study.
13. Previous therapy with approved or investigational tyrosine kinase or anti-VEGF receptor inhibitors or targeted therapies (e.g., multi-targeted kinase inhibitors such as sorafenib, AMG-706, sunitinib, pazopanib, lenvatinib).
14. RAI therapy within 12 weeks prior to first dose of study drug.
15. Radiation therapy other than RAI, including external beam, if not completed prior to randomisation.
16. For women: currently pregnant (confirmed with positive pregnancy test) or breast feeding.
17. Involvement in the planning and/or conduct of the study (applies to the Sponsor staff, its agents, and/or staff at the study site).

Supplementary Statistical Considerations

Patients in the efficacy analysis set with measurable disease at central review were considered for the analysis of BOR.

ORRs were compared between the two treatment groups using a logistic regression model adjusted for the following covariates: age, histology, WHO/ECOG PS, *BRAF* mutation status, and RET/papillary thyroid carcinoma (PTC) 1 and RET/PTC3 fusion status. The odds ratio was estimated together with associated two-sided CIs, *p* values were based on the likelihood ratio test, and CIs used a profile likelihood approach. The adjusted ORR is presented for each treatment group. ORRs derived according to the central review data are summarized by treatment group. Any patients who did not have measurable disease at baseline was excluded as per the RECIST 1.1 criteria.

The absolute values, change in target lesion TS from baseline, and percentage change in target lesion from baseline were summarized using descriptive statistics and were presented at each time point and by randomized treatment group. TS was based on the investigator's measurements of the target lesions. The percentage change from baseline at Week 36 in target lesion TS and the best percentage change from baseline were summarized and are presented by randomized treatment group. The number and percentage of patients in each treatment group with imputed Week 36 data are also presented. The effect of study treatment on percentage change in TS was estimated from an analysis of covariance (ANCOVA) model, including a term for the percentage change at Week 36 value and covariates for baseline TS, the time from the baseline scan to randomization, age (<65, ≥65 years), histology (papillary, follicular, other), WHO/ECOG PS (0, ≥1), *BRAF* mutation status (mutated, wild type, unknown/missing), and RET/PTC1 and RET/PTC3 fusion status (detected, not detected, unknown/missing). The results are presented in terms of adjusted means (least-square means weighted in line with the prevalence of covariates) for each term together with their associated two-sided CI. The difference in the least-square means and corresponding CI and *p* values are presented. TS is presented graphically using waterfall plots for each treatment group. Waterfall plots were also produced for the best percentage change from baseline in target lesion size. Missing or incomplete TS data are recorded as such. A windowing rule was applied and followed the protocol allowed visit window; therefore, any RECIST scan performed within ± 2 weeks of the protocol scheduled missed visit was used for that missing visit. If, after applying the above considerations to the missing data, there was still missing TS measurement, data collected from baseline and all visits up to

and including the first visit after week 36 were employed to fit a linear regression to generate an estimated value for TS measurement at week 36. If there was progression for an individual or of a non-target lesion, 20% TS increase at week 36 from baseline would be imputed. If data were completely not available, the patient would be excluded from analysis. In case of death, 20% TS increase at week 36 from baseline or largest increase calculated from actual or imputed data would be imputed.
The analysis of OS was performed using a log-rank test. The HR, corresponding adjusted two-sided CIs, and two-sided *p* value are presented. A Kaplan–Meier plot for OS is presented along with the median OS (with 95% CI), 25th and 75th percentiles, and the proportions of patients who were alive at 6 months, 1 year, and 2 years (with 95% CIs). Any patient not known to have died at the time of analysis was censored based on the last recorded date on which the patient was known to be alive. If there were no survival or death data available at the analysis data cut-off date, then the last date the patient is known to be alive will be calculated from the last assessment date of all modules (except the visit module) on the database and patients treated as censored patients at last recorded date.
The efficacy analysis set included all randomized patients regardless of whether they took randomized treatment or not. The safety analysis set instead included randomized patients who received at least one dose of treatment (vandetanib/placebo); indeed, 3 patients were excluded from the safety analysis set since they did not receive randomized treatment after enrolment (Supplementary Table S1).

Supplementary Subgroup Analysis

Log-rank unadjusted test showed that vandetanib treatment was associated with slight decreased risk of mortality (OS: HR 0.81; 95% CI 0.42-1.57) and slightly higher chances of PFS (HR 0.65; 95% CI 0.41-1.02) in patients <65 years of age. Vandetanib treatment was also associated with increased risk of mortality when the histology was papillary (OS: HR 1.04; 95% CI 0.60-1.80) or other (OS: HR 1.21; 95% CI 0.42-3.48) and slight decreased risk of mortality when the histology was follicular (OS: HR 0.88; 95% CI 0.36-2.11). Slight decreased risk of mortality and modest increased chances of PFS were also observed in vandetanib treatment group when RET/PTC1 (OS: HR 0.88; 95% CI 0.52-1.49; PFS: HR 0.66, 95% CI 0.45-0.99), RET/PTC3 (OS: HR 0.92; 95% CI 0.55-1.53; PFS: HR 0.67, 95% CI 0.46-0.99) and BRAF mutation (OS: HR 0.80; 95% CI 0.44-1.47; HR PFS: HR 0.63; 95% CI 0.40-1.00) were not detected.

SUPPLEMENTARY FIGURE S1. BOX PLOT OF ABSOLUTE THYROID-STIMULATING HORMONE VALUES IN THE RANDOMIZED TREATMENT PERIOD (SAFETY ANALYSIS SET)

Drg Dis., drug discontinuation.
Horizontal line, median; box, 25–75th percentile.

Whiskers extend to the most extreme observation within 1.5 times the interquartile range from the nearest quartile, so that all outliers >1.5 times the interquartile range and displayed individually.
