# Supplementary material for: Vandetanib in locally advanced or metastatic differentiated thyroid cancer refractory to radioiodine therapy
Source: Endocr Relat Cancer. 2024 Jul 2;31(8):e230354. doi: 10.1530/ERC-23-0354 (PMC11301419; doi:10.1530/ERC-23-0354)
Supplement: SUPPLEMENTARY TABLE S1. PATIENT DEMOGRAPHICS OF SUBJECTS ENROLLED, BUT THAT DISCONTINUED THE STUDY BEFORE RECEIVING RANDOMIZED TREATMENT [file supplementary_table_1.pdf]

SUPPLEMENTARY TABLE S1. PATIENT DEMOGRAPHICS OF SUBJECTS ENROLLED, BUT THAT DISCONTINUED THE STUDY BEFORE RECEIVING RANDOMIZED TREATMENT

	Subject 1	*Subject 2*	*Subject 3*
Age	59	76	69
Sex	Male	Male	Male
Race	White	White	White
Description of the planned arm	Placebo	Vandetanib 300 mg	Vandetanib 300 mg
Reason for discontinuation	Withdrawn from study due to subject decision	Withdrawn from study due to subject decision	Protocol deviation (no longer met eligibility criteria)

According to the study Statistical Analysis Plan, the subjects reported in table above were included in the efficacy analysis set, but excluded from the safety analysis set.
