# Supplementary material for: Vandetanib in locally advanced or metastatic differentiated thyroid cancer refractory to radioiodine therapy
Source: Endocr Relat Cancer. 2024 Jul 2;31(8):e230354. doi: 10.1530/ERC-23-0354 (PMC11301419; doi:10.1530/ERC-23-0354)
Supplement: SUPPLEMENTARY TABLE S2. SUMMARY OF EFFICACY IN PATIENTS WITH DIFFERENTIATED THYROID CANCER [file supplementary_table_2.pdf]

Supplementary Table S2. Summary of efficacy in patients with differentiated thyroid cancer

	Vandetanib 300 mg	*Placebo*
PFS (log-rank test, primary analysis)[a]	*n* = 119	*n* = 119
Patients with events,[b] *n* (%)	70 (58.8)	85 (71.4)
HR (95% CI)	0.75 (0.55–1.03)	
Two-sided *p* value	0.080	
Sensitivity of centrally reviewed PFS to assess possible ascertainment bias[a]	n = 119	n = 119
Patients with events,[b] *n* (%)	73 (61.3)	91 (76.5)
HR (95% CI)	0.75 (0.55–1.02)	
Two-sided *p* value	0.065	
PFS, Cox proportional hazards model adjusting for most recent TSH >0.1 mU/L[c]	*n* = 116	*n* = 118
Patients with events,[c] *n* (%)	68 (58.6)	85 (72.0)
HR (95% CI)	0.62 (0.44–0.87)	
Two-sided *p* value	0.005	
ORR, logistic regression	*n* = 119	*n* = 118
Number of patients with response (%)[d]	6 (5.0)	0 (0.0)
Adjusted response rate (%)[e]	NC	NC
Odds ratio[e]	NC	
95% CI[e]	NC, NC	
Two-sided *p* value	0.004	

Patients who did not have measurable disease at baseline are excluded from this table and the denominator in accordance with RECIST version 1.1 criteria.
Due to the small number of responders the logistic regression model including covariates as planned in the SAP was not feasible. A logistic regression model with a factor for treatment only was fitted instead.
An odds ratio >1 favors vandetanib 300 mg.
[a]The analysis was performed using an unadjusted log-rank test. HR <1 favors vandetanib 300 mg. Calculation of HR and CI follows (Berry G *et al.* 1991) and (Sellke and Sigmund 1983). Progression includes death in the absence of RECIST progression.

[b]Progression/death events that do not occur within 28 weeks of the last evaluable RECIST assessment (or randomization in the absence of an evaluable baseline RECIST assessment) are censored and therefore excluded in the number of events.
[c]The analysis was performed using a Cox proportional hazards model that included randomized treatment and the covariates age (<65 and ≥65 years), histology (papillary, follicular, other), WHO/ECOG performance status (0 and ≥1), *BRAF* mutation status, RET/PTC1 and RET/PTC3 fusion status (positive, not detected, unknown/missing), and a time-dependent binary covariate for most recent TSH >0.1 mU/L. HR <1 favors vandetanib 300 mg. Progression includes death in the absence of RECIST progression.
[d]Response does not require confirmation.
[e]NC due to 0 events in the placebo group.
CI, confidence interval; ECOG, Eastern Cooperative Oncology Group; HR, hazard ratio; NC, not able to calculate; ORR, objective response rate; PFS, progression-free survival; PTC, papillary thyroid carcinoma; RECIST, Response Evaluation Criteria in Solid Tumors; RET, rearranged during transfection; SAP, statistical analysis plan; TSH, thyroid-stimulating hormone; WHO, World Health Organization.

References

1. Berry G, Kitchin RM & Mock PA 1991 A comparison of two simple hazard ratio estimators based on the logrank test. *Statistics in Medicine* **10** 749-755.
2. Sellke T & Siegmund D 1983 Sequential analysis of the proportional hazards model. *Biometrika* **70** 315-326.
